# Central Breast Excision With Immediate Autologous Reconstruction for Recurrent Periductal Sepsis: An Application of Oncoplastic Surgical Techniques

**Published:** 2012-08-01

**Authors:** Sinclair M. Gore, Gordon C. Wishart, Charles M. Malata

**Affiliations:** ^a^Department of Plastic Surgery; ^b^Cambridge Breast Unit, Addenbrooke's Hospital, Cambridge University Hospitals NHS Trust, Cambridge, CB2 2QQ, United Kingdom

## Abstract

**Objective:** The aim of this procedure was to definitively treat periductal mastitis and periareolar sepsis which was previously resistant to multiple surgical procedures and nonoperative treatment of chronic nipple sepsis. **Methods:** We employed a multidisciplinary approach to the treatment of end-stage periductal mastitis using a combination of central breast excision and immediate autologous latissimus dorsi flap reconstruction. **Results:** Clearance of periductal mastitis and infection has been achieved with no recurrence at 3 years. Good symmetry of breast shape and volume has been achieved using this technique. **Conclusions:** This method of partial breast reconstruction, commonly used for reconstruction of breast cancer ablative defects, may also provide good outcomes in nonmalignant disease.

Periductal mastitis and duct ectasia are benign breast conditions associated with periareolar sepsis^1^ that often present with multiduct, multicolored nipple discharge. Periductal mastitis appears to be more common in smokers and tends to occur in younger women; in extreme cases, it can be responsible for development of nonlactational abscesses and fistulae. This can be troublesome for affected patients as the recurrent sepsis is often resistant to nonoperative intervention. Furthermore, it can be very difficult to exclude malignancy in these cases and triple assessment using clinical breast examination, imaging, and biopsy may still miss a small proportion of cases.^2^ The presence of blood-stained nipple discharge carries a greater risk of underlying malignancy^2^^,^^3^ and diagnostic surgery, either by microdochectomy or total duct clearance, is required.

The management of persistent multiduct nipple discharge often requires surgery, especially when it is associated with periareolar sepsis and interferes with quality of life. The best treatment is often total duct excision (Hadfield's operation), but this procedure does carry recognized morbidity and can be complicated by recurrent sepsis in certain cases, especially in smokers.^4^ These patients often have recurrent symptoms despite multiple operations and can be extremely difficult to manage.

We report a multidisciplinary approach to the treatment of end-stage periductal mastitis using a combination of central breast excision and immediate autologous latissimus dorsi (LD) flap reconstruction.

## SURGICAL METHOD

**Figure 1:**
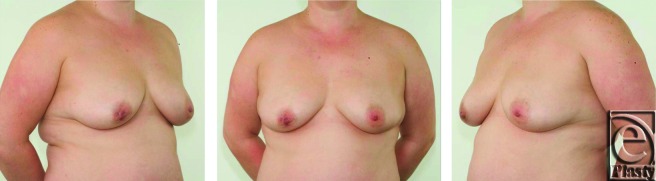
Preoperative appearance of a 38-year-old patient with grade 2 ptotic breasts.

**Figure 2:**
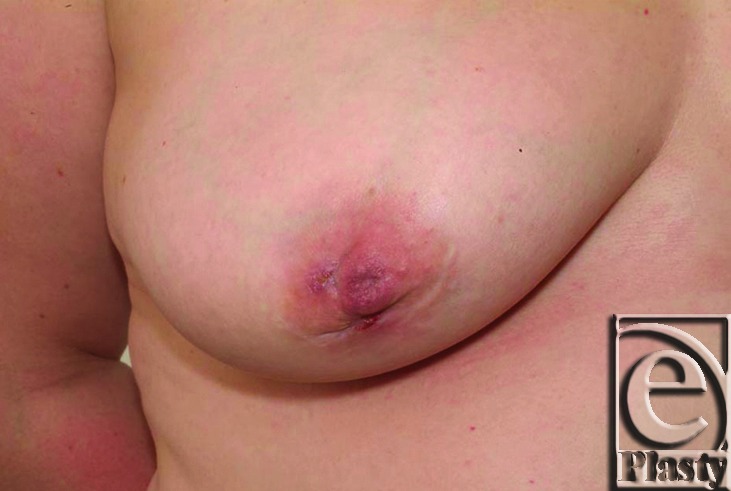
Close-up view of right breast. Note the distortion of the right nipple and areola.

A 38-year-old lady was referred to our regional breast unit from a local district hospital with recurrent nipple discharge and fistula formation in the right breast. She had had 4 previous duct excision operations on the right breast and 1 limited duct excision on the left breast (with no further problems on that side). Noticeable nipple distortion and asymmetry was present due to multiple procedures to the right breast (Figs 1 and 2).

Following initial breast examination and radiological assessment, it was considered that further local surgery was likely to be unsuccessful and the possibility of central breast excision, combined with immediate autologous reconstruction of the tissue defect was discussed with the patient. The patient was keen to proceed with this option, and surgery was planned by the breast and reconstructive surgeons.

At surgery, a central breast excision, or limited “cylinder” mastectomy, was performed including the nipple-areolar complex (NAC) and all the breast tissue deep to this down to the level of the pectoral fascia. The specimen measured 4.5 × 4.5 × 6.5 cm in maximum dimension and weighed 45 g. A tunnel was then created deep to the breast tissue in the upper outer quadrant of the breast and extended subcutaneously into the axilla to allow delivery of the tissue flap to the central part of the breast. This was facilitated by “shaving” the under surface of the breast tissue in this area. Immediate autologous tissue reconstruction, using a pedicled LD myocutaneous flap, was performed to allow replacement of the volume deficit and skin defect following excision of the NAC. The surgery was performed synchronously by breast and reconstructive surgeons under antibiotic cover, with the patient lying on her left side, as is done for mastectomy and immediate breast reconstruction. Following division of the thoracodorsal nerve (to denervate the muscle and reduce its bulk) and trimming of the proximal muscle the flap was transposed anteriorly to the breast defect, with the flap pedicle lying deep to the superolateral breast tissue. The muscle was “swiss-rolled” with 2/0 PDS sutures to create a cylindrical mass. The skin paddle of the myocutaneous flap was adjusted to the size of the resected NAC with the lateral wings de-epithelialized, folded over, and secured to the cylindrical walls of the defect. The skin island was then inset at a dermal level with 3/0 Monocryl. Antibiotic cover was continued for a further 5 days. Nine months following this procedure, bilateral breast augmentation was undertaken using anatomically shaped breast implants. A further 10 months following this, right nipple reconstruction (modified C-V flap technique) was performed. Finally, nipple-areola complex tattooing was employed to optimize breast symmetry.

## SURGICAL RESULT

**Figure 3:**
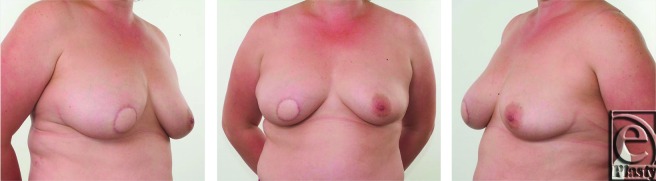
Views following latissimus dorsi flap reconstruction. Note the preservation of the breast contour and volume.

**Figure 4:**
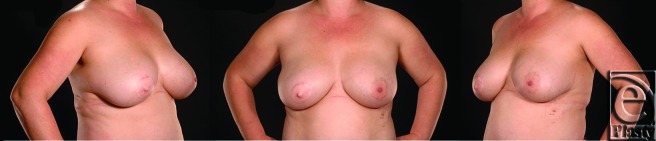
Final result 24 months following initial resection and latissimus dorsi flap reconstruction, following adjunctive bilateral breast augmentation, right nipple reconstruction, and nipple-areola complex tattooing. Note the final breast symmetry attained.

**Figure 5:**
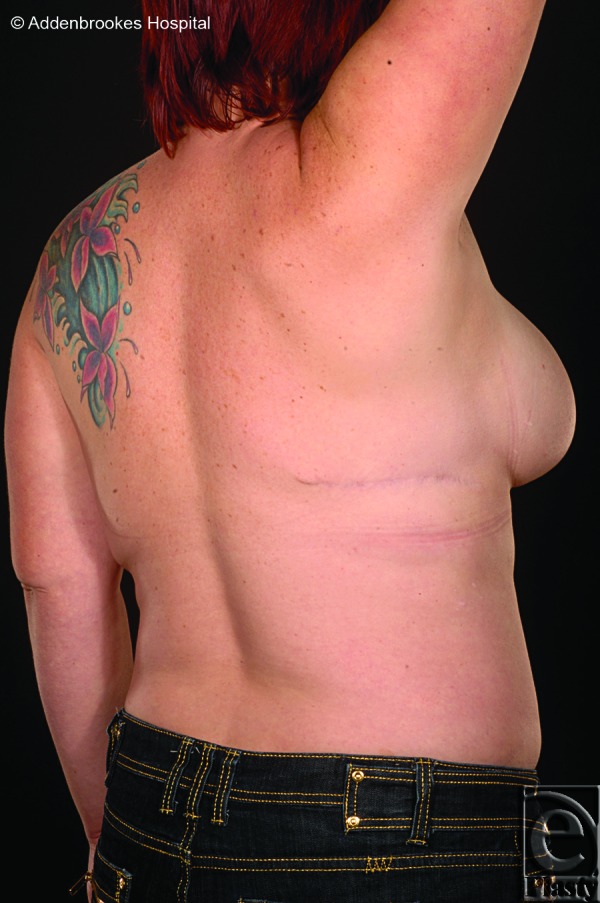
Well-healed latissimus dorsi flap donor site.

Results of the first reconstruction after 3 months are shown in Figure 3. The final result following the adjunctive procedures (more than 2 years after initial resection and LD flap reconstruction) is shown in Figure 4. Good symmetry of breast form and volume has been achieved. The patient is pleased with this result and has not suffered any from any infection, problems with wound healing or recurrence of periductal sepsis. The appearance of the well-healed donor site scar is shown in Figure 5.

## DISCUSSION

The technique performed here borrows heavily from oncoplastic procedures used to reconstruct central lumpectomy and central quadrantectomy (partial mastectomy) defects during breast-conserving surgery for cancer.^5^^,^^6^ Its application to benign disease, however, is novel, although it follows the same principles. A “therapeutic mammaplasty” breast parenchymal rearrangement was thought not to be ideal in the presence of indolent or repeated sepsis associated with induration of the breast tissue and persistent inflammation. It was also precluded, as the patient did not wish to have smaller breasts or contralateral surgery with a lot of scars in the form of a mastopexy. Having ruled out such volume displacement procedures, it was decided to employ the versatile volume replacement technique of LD myocutaneous flap reconstruction.^7^^,^^8^

Its particular advantages in this setting include the long pedicle reach to the central part of the breast, the replacement of the areola by the skin paddle of the flap, and the replacement of parenchymal volume by the (“swiss-rolled”) muscle. In addition, the de-epithelialized wings of the skin paddle were folded laterally to provide both firm anchorage to the breast parenchyma and soft tissue volume, which will endure long after muscle atrophy has occurred. Further dissection within the glandular breast tissue (and therefore the potential risk of intrabreast sepsis) was avoided by not rearranging breast parenchyma, but dissecting in a plane deep to the breast, enabling flap transfer at a level superficial to the thoracic wall.

The potential disadvantages of this technique include the excess tissue bulk in the axilla and a tight tunnel under the lateral breast. In this case, both issues were addressed by trimming the part of the muscle proximal to entry of the vascular pedicle and by “shaving” the overlying parenchyma of the lateral breast above the tunnel. Like the Nottingham group,^9^ we divided the tendon and fully mobilized the vascular pedicle to enable the flap to be sited almost exclusively within the breast. However, we also recommend additional soft tissue anchoring besides the skin inset and prior trimming of the proximal muscle (especially in thin women).

Inclusion of LD muscle within the flap gave added bulk to the reconstruction and provided safe vascular supply to the skin paddle in the absence of a reliable single thoracodorsal artery perforating vessel. Although atrophy is to be expected in such a setting, a problem with progressive deterioration in symmetry was not noted in this case. Volume enhancement by means of bilateral breast augmentation 9 months following initial surgery has reduced the margin for asymmetry and the final results shown over 2 years from initial surgery show a stable symmetrical result with which the patient is satisfied.

The initial color mismatch of the flap skin paddle has been overcome by nipple reconstruction and NAC tattooing. Although immediate nipple reconstruction has been advocated by others,^9^ we preferred to defer this because of the potential risks of infection and healing problems.

Although the LD flap has been extensively used for reconstructing volume loss defects resulting from cancer (mastectomy, quadrantectomy, and lumpectomy), traumatic and congenital disorders, this is, to our knowledge, its first use for reconstruction following benign disease due to recalcitrant infection.

## CONCLUSION

We recommend that in end-stage cases of periductal mastitis and fistula formation radical resection be considered to reduce the risk of recurrent sepsis. This can be performed in conjunction with immediate partial breast reconstruction with excellent results, in this case performed as a synchronous procedure by breast and reconstructive surgeons. Subsequent symmetrizing procedures can optimize the final result. In this case, we have attained a high level of patient satisfaction with no complications or recurrence of the underlying disease process.
